# Targeting the gut microbial metabolic pathway with small molecules decreases uremic toxin production

**DOI:** 10.1080/19490976.2020.1823800

**Published:** 2020-10-04

**Authors:** Yingyi Wang, Jianping Li, Chenkai Chen, Jingbo Lu, Jingao Yu, Xuejun Xu, Yin Peng, Sen Zhang, Shu Jiang, Jianming Guo, Jinao Duan

**Affiliations:** a Jiangsu Collaborative Innovation Center of Chinese Medicinal Resources Industrialization, Nanjing University of Chinese Medicine, Nanjing, China; b Jiangsu Key Laboratory for High Technology Research of TCM Formulae, Nanjing University of Chinese Medicine, Nanjing, China.

**Keywords:** Gut microbiota, chronic kidney disease, uremic toxin, indoxyl sulfate, indole, isoquercitrin

## Abstract

Uremic toxins are a class of toxins that accumulate in patients with chronic kidney disease (CKD). Indoxyl sulfate (IS), a typical uremic toxin, is not efficiently removed by hemodialysis. Modulation of IS production in the gut microbiota may be a promising strategy for decreasing IS concentration, thus, delaying CKD progression. In the present study, we identified isoquercitrin (ISO) as a natural product that can perturb microbiota-mediated indole production without directly inhibiting the growth of microbes or the indole-synthesizing enzyme TnaA. ISO inhibits the establishment of H proton potential by regulating the gut bacteria electron transport chain, thereby inhibiting the transport of tryptophan and further reducing indole biosynthesis. This non-microbiocidal mechanism may enable ISO to be used as a therapeutic tool, specifically against pathologies triggered by the accumulation of the microbial-produced toxin IS, as in CKD. Herein, we have shown that it is possible to inhibit gut microbial indole production using natural components. Therefore, targeting the uremic toxin metabolic pathway in gut bacteria may be a promising strategy to control host uremic toxin production.

## Graphical abstract

**Figure:**
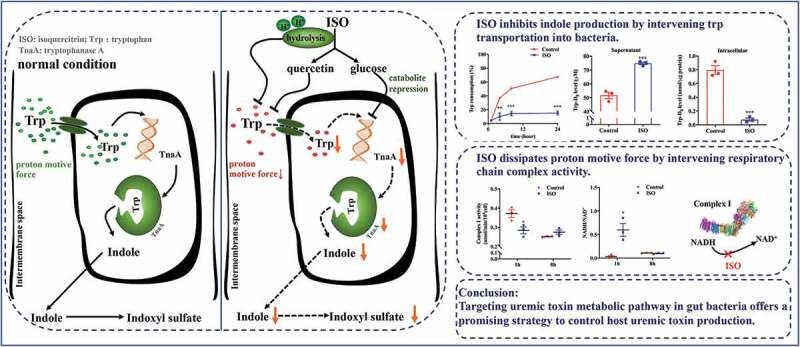

## Introduction

In recent years, the drugging of the gut microbiota has been proposed as a promising strategy for the treatment of different human diseases. In 2015 and 2018, Hazen and colleagues identified the first small-molecule antagonist for the critical initiating microbial TMA lyase step in atherosclerosis development and thrombosis potential.^1,2^ In 2018, Jiang and colleagues reported that metformin acts in part through the gut microbiota (*Bacteroides fragilis*–GUDCA–intestinal FXR axis) to improve metabolic dysfunction.^3^ Their studies showed that targeting the gut bacterial metabolic pathway may be a promising strategy for the treatment of human disease.

Chronic kidney disease (CKD), with a total global prevalence of 10%, has become a significant public health issue.^4–6^ Indoxyl sulfate (IS), a typical protein-bound uremic toxin molecule produced by gut bacteria, is normally cleared from the body via renal excretion. This microbial-derived metabolite accumulates in the body when kidney function decreases. The concentrations of IS are normally maintained at 10– 130 mg/day in healthy individuals.^7^ However, in dialysis patients with end-stage renal disease, circulating IS levels are 20 times higher than normal.^8,9^

IS not only acts as a biomarker for CKD, but also serves as an active participant in CKD pathogenesis.^10,11^ In rodent animal models, administration of IS induces the expression of genes related to renal tubulointerstitial fibrosis and glomerular sclerosis, as well as reduced renal function.^12^ In addition to being a uremic toxin, IS has attracted considerable attention as a risk factor for cardiovascular dysfunction in CKD patients,^13,14^ and high serum IS levels are correlated with metabolic and cardiovascular disease in people with CKD.^15–17^

Accumulating evidence has shown that CKD progression can be delayed by decreasing the circulating levels of IS.^18–20^ The clinical treatment of end-stage renal disease has traditionally focused on the removal of toxins by dialysis, while alternative methods that inhibit uremic toxin production have not attracted much attention. Therefore, interventions that can decrease IS production are of considerable interest for their potential therapeutic benefit for the treatment of CKD and related cardiovascular diseases.

In mammals, IS formation occurs via a three-step meta-organism pathway. Following nutrient ingestion, indole can be formed from dietary tryptophan by intestinal bacteria tryptophanases (TnaA).^21^ Following absorption into the portal vein, indole is metabolized to indoxyl and then sulfated to IS in the host liver.^22^ Microbial tryptophan catabolites are important for homeostasis in the gut in non-CKD settings. These metabolites are known to have beneficial effects on host health. Indole has been shown to activate the immune system through the aryl hydrocarbon receptor.^23^ Furthermore, indole also strengthens intestinal epithelial barrier,^24^ regulates enteroendocrine cell secretion,^25^ reduces intestinal inflammation, and stimulates intestinal motility. The latest evidence suggests that indole is relevant to human nonalcoholic fatty liver disease, and can significantly reduce the severity of liver steatosis and inflammation.^26^

In terms of CKD, indole, a critical precursor of IS, is harmful to the body. Therefore, one appropriate approach to decrease IS levels is to reduce indole production in the gut. AST-120, a kind of absorbent, has been reported to absorb indole in the gut, thus, decreasing the amount of absorbed indole leading to decreased IS concentrations and delayed CKD progression.^27,28^ These reports have proven that decreasing gut indole production may be an attractive and promising strategy for delaying CKD progression. However, modulation of the gut microbial metabolic pathway and indole production by small molecules has not yet been reported.

Isoquercitrin (quercetin-3-O-D-glucopyranoside; ISO) is a monoglucoside of the most abundant natural flavonoid, quercetin, and is widely distributed in herbs, fruits, vegetables, and plant-source beverages such as wine and tea.^29,30^ In the present study, we found that ISO, a small molecule flavonoid, can modulate the production of indole in gut bacteria through regulation of the gut bacteria electron transport chain.

## Results

### ISO inhibits indole formation in gut single bacterial cultures and polymicrobial cultures

As an intercellular signaling molecule, indole is produced by the degradation of tryptophan by a large number of gram-positive and gram-negative bacteria.^31^ Early researchers found that *Escherichia coli* could produce and secret indole into the culture supernatant.^32^ In addition, studies have shown that the abundance of Enterobacteriaceae in the gut of CKD mice is significantly increased. Therefore, we chose *E. coli* as a model bacterium to examine whether the presence of ISO could inhibit indole production in gut bacterial cultures. *E. coli* cultures were incubated with 50 μM, 100 μM, and 200 μM ISO for 1, 2, 4, 8, 20, and 24 h, followed by measuring the concentrations of indole in intracellular and supernatants using High performance liquid chromatography-fluorescence detector (HPLC-FLD). The effect of ISO on bacterial growth was also evaluated by measuring the optical density at 600 nm (OD_600nm_).

**Figure 1.**
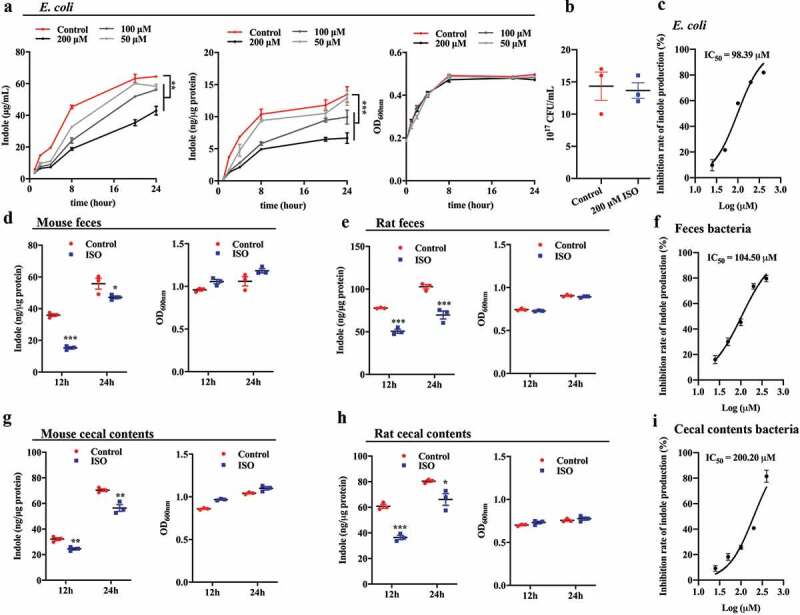
ISO significantly inhibits the production of indole by intestinal bacteria from different sources. (a) ISO used at 50, 100, and 200 μM inhibited the production of indole in *E. coli* in a dose-dependent manner. Red line: control group, black line: 200 μM ISO-treated group, dark gray line: 100 μM ISO-treated group, and light gray line: 50 μM ISO-treated group. Red point: control group, blue block: 200 μM ISO-treated group. (b) Colony-Forming Unit/ml (CFU/ml) of *E. coli* after an 8 h treatment with or without 200 μM ISO. Dose response curves and IC_50_ of indole production inhibition rate after (c) *E. coli*, (f) feces mixed bacteria and (i) cecal mixed bacteria co-incubated with ISO (25, 50, 100, 200, 400 μM) for 8 h. At 200 μM, ISO inhibited indole production in mixed intestinal bacterial samples collected from (d) mouse feces, (e) rat feces, (g) mouse cecal contents and (h) rat cecal contents. All the data were normalized with OD or intracellular protein. *P < .05, **P < .01, ***P < .001 vs. control group.

We found that ISO significantly inhibited *E. coli* indole production in a concentration dependent manner (Figure 1a), both in intracellular and supernatant samples taken from the bacterial cultures. Importantly, ISO was non-bactericidal and non-bacteriostatic (i.e. no antibiotic activity), as it did not inhibit bacterial growth (Figure 1b). ISO does not have a general effect on *E. coli* growth, but it does affect the ability of *E. coli* to produce indole. The IC_50_ of ISO for indole synthesis inhibition is 98.39 μM (Figure 1c). Thereafter, we explored the impact of ISO on indole production and bacterial growth using mixed bacteria. We chose mouse/rat cecal contents and feces as physiological polymicrobial sources to test the inhibitory effect of ISO on indole production. To obtain fecal mixed bacteria and cecal mixed bacteria we collected cecal contents and feces from C57BL/6 mice and Sprague-Dawley (SD) rats. The polymicrobes were then treated with 200 μM ISO and anaerobically incubated for 12 h and 24 h. ISO significantly inhibited indole production in gut polymicrobial cultures, but it did not inhibit the growth of bacteria (Figure 1d, 1e 1 g, and 1h). The IC50 of ISO on indole synthesis is 104.50 μM and 200.20 μM in fecal bacteria and cecal contents bacteria, respectively (figure 1(f,i)). These results showed that the ability of ISO to inhibit indole production in complex microbial communities is lower than that of single organism.

### ISO does not directly inhibit tryptophanase activity but reduces exogenous tryptophan transport into bacteria

Indole is generated by TnaA, which can hydrolyze tryptophan into indole, pyruvate, and ammonia^33^ in the tryptophan pathway.^21^ Thus far, TnaA is the only enzyme known to produce indole in bacteria, and TnaA-catalyzed indole production is done entirely within the bacteria.^31^

**Figure 2.**
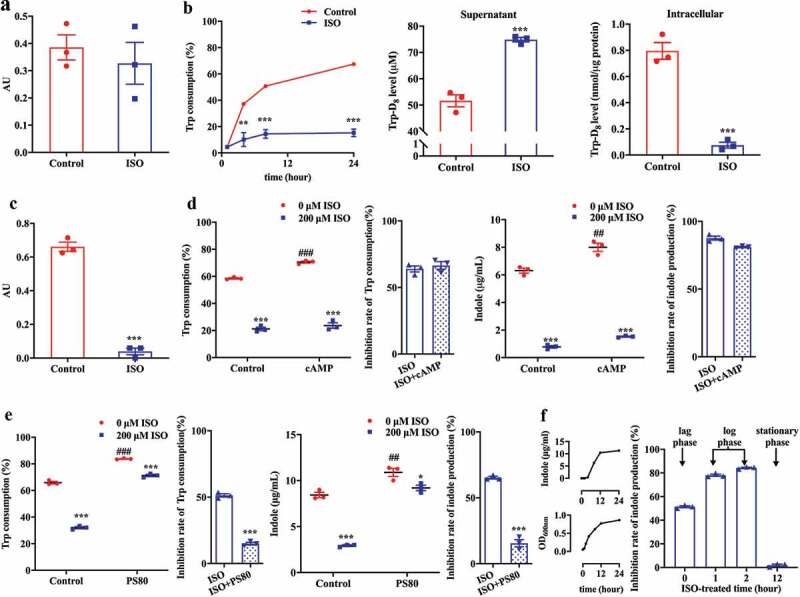
ISO does not directly inhibit tryptophanase activity but reduces exogenous tryptophan transport into *E. coli*. (a) Effect of ISO on TnaA activity. AU = 1 μg indole produced/min/OD_600nm_ of bacteria. (b) Effect of ISO on tryptophan consumption (left), and the change in concentration of isotope labeling DL-tryptophan-D_8_ in the intracellular fraction (middle) and supernatant (right) after an 8 h treatment with 200 μM ISO. Tryptophan (Trp) consumption (100%) was calculated as [initial tryptophan (μM) – supernatant tryptophan (μM)]/initial tryptophan concentration (μM). (c) Inhibitory effect of ISO on intracellular TnaA enzyme content. AU = 1 μg indole produced/min/OD_600nm_ of bacteria. (d) The influence of the absence or presence of cAMP (5 mM, 8 h) on the effects of ISO on tryptophan consumption (left) and indole production (right). (e) Effect of ISO on tryptophan consumption (left) and indole production (right) in *E. coli* after treatment with 0.5% PS80 for 8 h. (f) Anaerobic growth of *E. coli* and its indole-production curve (left), inhibition rates of indole biosynthesis in *E. coli* following treatment with ISO during different phases of growth (right). All the data were normalized with OD or intracellular protein. *P < .05, **P < .01, ***P < .001 vs. control group under the same experimental conditions. ^##^ *P* < .01, ^###^ *P* < .001 vs. control group (0 μM ISO).

We hypothesized that ISO might inhibit TnaA enzyme activity, thereby decreasing TnaA-catalyzed indole production. To test this hypothesis, TnaA was extracted from *E. coli*, incubated with 200 μM ISO, and we determined the enzyme activity using an enzymatic reaction assay. We found that TnaA enzyme activity was not affected by ISO (*P* >.5; Figure 2a), thereby suggesting that ISO does not directly inhibit TnaA enzyme activity and does not interfere with the transformation of tryptophan to indole.

In gut bacteria, indole production generally depends on the presence of exogenous tryptophan, which is taken up into bacteria and converted into indole by TnaA.^33^ As the uptake of tryptophan into gut bacteria is another key pathway for indole production, we postulated that ISO may intervene in the transportation of tryptophan into gut bacteria. Luria-Bertani (LB) medium contains approximately 0.51 mM tryptophan. After bacteria were treated with ISO for 1, 4, 8, and 24 h, we found that tryptophan consumption was significantly lower compared to the control group at 4– 24 h (Figure 2b). To further confirm the inhibitory effect of ISO on tryptophan consumption, the concentration of intracellular and extracellular (i.e. in the culture media) isotope labeled DL-tryptophan-D_8_ (Trp-D_8_) was determined by Ultra-performance liquid chromatography coupled with triple-quadrupole tandem mass spectrometry (UPLC-TQ/MS). In this assay, 0.02 mL Trp-D_8_ (5 mM) was added to 0.48 mL LB medium (the final concentration of Trp-D_8_ was 200 μM) which was then incubated with *E. coli* and 200 μM ISO for 8 h. In ISO group, the intracellular Trp-D_8_ level was 0.08 nmol/μg protein, which was lower compared with control group (0.80 nmol/μg protein). Similarly, in ISO group, Trp-D_8_ level in supernatant was 74.86 μM, which was higher compared with control group (51.64 μM). This result suggested that ISO reduced the transport of tryptophan into bacteria.

Under physiological conditions, when exogenous tryptophan is transported into bacteria, the presence of intracellular tryptophan induces *TnaA* gene expression and TnaA protein synthesis. Therefore, if exogenous tryptophan uptake was inhibited by ISO, intracellular TnaA protein amount would be downregulated. Following treatment with ISO, intracellular TnaA levels in *E. coli* decreased significantly (Figure 2c). These data confirm that ISO intervenes in tryptophan consumption and decreases intracellular TnaA levels, thereby decreasing indole production.

The second messenger cyclic adenosine monophosphate (cAMP) can amplify and transduce signals in bacteria.^34^ In *E. coli*, the cAMP receptor protein (CRP) is activated after binding with cAMP, the CRP-cAMP complex regulates the transcription of *TnaA* and *TnaB*.^35^ Exogenous cAMP (5 mM) promotes tryptophan consumption and indole production, which is likely due to increased *TnaA/B* gene expression and TnaA/B protein synthesis (Figure 2d). However, in the presence of cAMP, ISO still significantly inhibits tryptophan consumption and indole synthesis, thereby suggesting that ISO does not directly act on *TnaA* gene expression and, thus, TnaA protein synthesis in *E. coli*.

Tryptophan is transported by TnaB permease, which is found in the bacterial plasma membrane and plays an important role in the transport of tryptophan. Polysorbate-80 (PS80), a nonionic surfactant, increases the permeability of the bacterial membrane.^36,37^ Therefore, in the presence of PS80, tryptophan directly passes through the bacterial membrane without the aid of TnaB permease. Accordingly, we hypothesized that in the presence of PS80 more tryptophan molecules would enter the bacteria, leading to attenuation of the inhibitory effect of ISO on tryptophan transport. As we postulated, tryptophan intake increased when 0.5% (v/v) PS80 was added to the LB medium (Figure 2e). As a consequence, the inhibitory effect of ISO on indole production and tryptophan consumption decreased after treatment with 0.5% (v/v) PS80 for 8 h.

Furthermore, we compared the inhibitory ability of ISO on indole production for different bacterial growth phases. After incubation for 1 h, bacterial growth fell into the log phase with significantly increased indole production (figure 2f). Co-incubation with log phase bacteria for 10 h resulted in approximately 80% inhibition of ISO. When ISO was co-incubated with lag phase bacteria, the inhibition rate decreased to 50%. The inhibitory effect of ISO on indole-production almost completely disappeared when co-incubated with stationary phase bacteria for 10 h. These experiments supported the hypothesis that ISO represses tryptophan transport into bacteria.

### ISO dissipates proton motive force by intervening in respiratory chain complex activity

**Figure 3.**
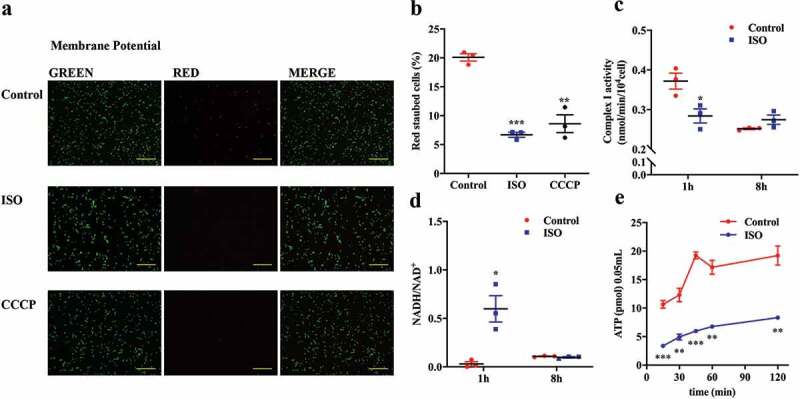
ISO dissipates proton motive force by intervening in respiratory chain complex activity. (a) Representative images: effect of ISO and membrane potential dissipation agents on DiOC_2_(3)-uptake in *E. coli* after anaerobic incubation for 1 h. The upper panel shows untreated *E. coli* with normal membrane potential. The effects of treatments with 200 μM ISO and 10 μM CCCP are shown in the middle and lower panels, respectively. Scale bars: 500 μM. (b) Quantitative results of bacterial membrane potential in each group. (c) The activity of respiratory chain complex I in *E. coli* after incubation with 200 μM ISO for 1 or 8 h. (d) NADH/NAD^+^ ratio in *E. coli* after incubation with 200 μM ISO for 1 and 8 h. (e) Intracellular ATP content in *E. coli* after incubation with 200 μM ISO for 0 to 120 min. Experiments A to D were performed under anaerobic conditions. *P < .05, **P < .01, ***P < .001 vs. control group under the same experimental conditions.

The transport of exogenous tryptophan into bacteria requires the synergism of proton-motive force, which is generated by electron transport chains embedded in the bacterial membrane.^38^ Thus, we were curious to understand whether ISO inhibits tryptophan transport by decreasing the proton-motive force. We used fluorescence microscopy and a membrane potential probe, carbocyanine dye DiOC_2_ (3; 3,3ʹ-Diethyloxacarbocyanine Iodide) to analyze the cell membrane potential. At low concentrations, DiOC_2_(3) emits a green fluorescence in bacterial cells, but it becomes concentrated in cells with a normal membrane potential, causing the dye to self-associate and the fluorescence emission to shift to red. Freshly incubated *E. coli* cells exhibit medium-red staining by DiOC_2_(3), indicating a normal state of membrane potential (Figure 3(a,b)). After treatment with ISO for 1 h, bacteria showed weaker red fluorescence intensity, indicating decreased membrane potential. According to the ImageJ analysis, the proportion of red fluorescence intensity decreased from 20.08% to 6.69%. Similar results were obtained when bacteria were treated with the membrane potential dissipating agent carbonyl cyanide 3-chlorophenylhydrazone (CCCP) for 5 min.

According to the chemiosmotic hypothesis, electrons flow through the respiratory chain embedded in the *E. coli* membrane and form a proton gradient. Complex I is an important complex in the respiratory chain and can generate proton potential.^39^ The activity of bacterial membrane complex I was evaluated at 1 h or 8 h after incubation with ISO. Bacterial respiratory chain complex I activity decreased when *E. coli* was incubated with ISO for 1 h (Figure 3c).

NADH-ubiquinone oxidoreductase (complex I) is the core point for electrons to pass through the *E. coli* respiratory chain.^40,41^ Complex I is important for the formation of the proton motive force in the bacterial membrane. ComplexⅠcouples electron transfer from NADH to ubiquinone with the transport of four protons across the inner membrane. Complex I converts NADH to NAD^+^ and protons, and the electrons liberated from this reaction are shuttled to the respiratory chain. Thus, the NADH/NAD^+^ ratio is another way to reflect complex I activity. If complex I activity is inhibited, the NADH/NAD^+^ ratio will increase because less NADH is catalyzed to NAD^+^. Consistent with results obtained on complex I activity, the NADH/NAD^+^ ratio increased significantly after *E. coli* was treated by ISO for 1 h (Figure 3d).

As the energy source in bacteria, adenosine triphosphate (ATP), is synthesized by ATP synthase, which is driven by a proton gradient. The complexes of the electron transfer system generate a proton potential, which drives ATP synthesis through ATP synthase (complex V).^39^ Thus, if ISO interferes with the proton gradient, ATP synthesis would decrease accordingly. To test this hypothesis, bacterial intracellular ATP was measured between 15 min and 2 h after co-incubation of *E. coli* and ISO (Figure 3e). ISO treatment significantly decreased ATP content, thereby supporting the hypothesis that ISO interferes with the proton gradient and consequently intervenes in tryptophan transport.

Collectively, these experiments concur with the theory that ISO dissipates proton motive force by interfering with respiratory chain complex activity, which leads to reduced ATP production.

In principle, *E. coli* can utilize several terminal electron acceptors, including oxygen, trimethylamine N-oxide (TMAO), and dimethyl sulfoxide (DMSO) *in vitro*. Under aerobic conditions, oxygen is the terminal electron acceptor. In the anaerobic state, TMAO and DMSO are external terminal electron acceptors in the respiratory chain.^42,43^ In the presence of terminal electron acceptors, alternative electrons will be supplied, which will compensate for the ISO disturbed proton motive force dissipating effect. Therefore, terminal electron acceptors may attenuate the ISO induced proton motive force dissipating effect and indole production inhibition effect.

**Figure 4.**
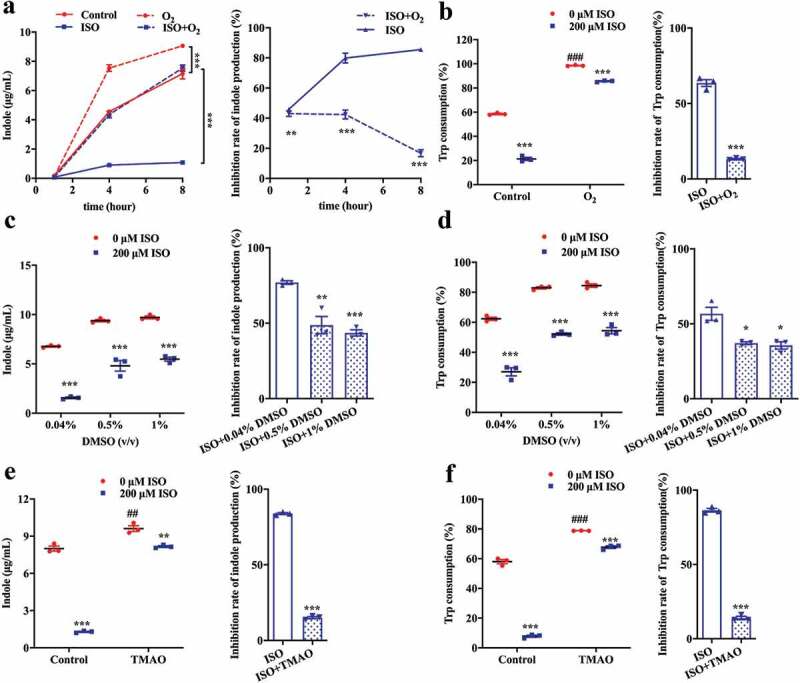
The electron receptor attenuates the inhibitory effect of ISO on indole production. (a) *E. coli* and 200 μM ISO were co-incubated aerobically for 1, 4 and 8 h. Indole concentration in the culture supernatant (right) and the inhibition rate of indole production (left) are presented. (b) Tryptophan consumption (right) and the inhibition rate (left) in *E. coli* after incubation with 200 μM ISO for 8 h. (c and d) The influence of electron receptor, DMSO (0.5% or 1%), on indole production (c) and tryptophan consumption (d) and the corresponding inhibition rates in *E. coli* after incubation with 200 μM ISO for 8 h. (e and f) The influence of electron receptor, TMAO (40 mM), on indole production (e) and tryptophan consumption (f) and the corresponding inhibition rates in *E. coli* after incubation with 200 μM ISO for 8 h. All the data were normalized with OD. *P < .05, **P < .01, ***P < .001 vs control group under the same experimental conditions. ^##^ *P* < .01, ^###^ *P* < .001 vs. control group (0 μM ISO).

As shown above, under anaerobic conditions, ISO continuously inhibits indole synthesis in *E. coli* (Figure 4a), with 51.8%, 80.32%, and 85.62% inhibition when the cells were cultured for 1, 4, and 8 h, respectively. When *E. coli* was incubated under aerobic conditions, more electrons were generated and a greater proton motive force was produced due to the presence of oxygen, which facilitated tryptophan consumption and indole synthesis (Figure 4(a,b)). Consequently, oxygen attenuates the inhibitory activity of ISO on tryptophan transport and indole production.

Similarly, in the presence of the electron receptors DMSO and TMAO, tryptophan consumption and indole production were also enhanced (Figure 4c, 4d, 4e, and 4f). As a result, DMSO and TMAO attenuated the inhibitory activity of ISO on tryptophan consumption and indole production.

Collectively, these data suggest that electron receptors attenuate the inhibitory potency of ISO on tryptophan consumption and indole production.

### ISO inhibits indole and IS production in vivo

**Figure 5.**
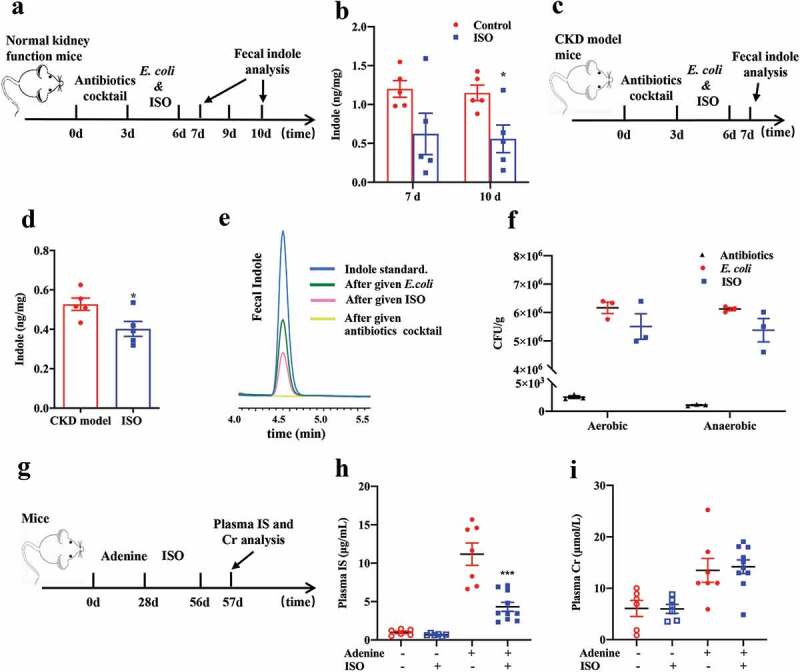
ISO inhibits indole and IS production *in vivo*. Schematic diagram of the experimental design and the indole content in mice feces after intestinal microbiota transplantation in (a and b) normal kidney function mice and (c and d) adenine-induced CKD model mice (n = 5 in each group). In Figure 5a, indole concentration was detected in day 7 and day 10, and the corresponding data was shown in Figure 5b. In Figure 5c, indole concentration was detected in day 7, and the corresponding data was shown in Figure 5d. *P < .05 vs. normal kidney function or CKD model group under the same experimental conditions. (e) Representative HPLC chromatogram of feces indole in each stage of this experiment. Light green, after given antibiotics cocktail to mice for 3 d; green, after given *E. coli* to mice for 3 d; pink, after given 80 mg/kg ISO to mice for 3 d or 6 d; blue, indole standard. (f) After antibiotics cocktail treatment and *E. coli* colonization, the colony-forming of mixed bacteria from mice feces mixed bacteria in aerobic or anaerobic conditions. Schematic diagram of the experimental design (g) and the plasma IS (h) and Creatinine (i) concentration of mice in each group after treatment with 80 mg/kg ISO for 4 weeks. Cr: Creatinine. Normal kidney function mice with or without ISO: n = 6, CKD model (adenine-treated) mice with or without ISO: n = 10. ***P < .001 vs CKD model (adenine-treated).

To validate our *in vitro* findings, we investigated the inhibitory effect of ISO on indole and IS production *in vivo* using intestinal microbiota transplantation to simulate the rapid growth of bacteria in C57BL/6 mice. After removing the gut microbiota of normal mice and adenine-induced CKD model mice with an antibiotic cocktail, mice gut bacteria were reconstructed with *E. coli* for 3 days, and then mice were treated with ISO (80 mg/kg) by oral gavage for 6 days (normal mice) or 3 days (CKD model mice) (Figure 5a~5e). After ISO treatment, we found that fecal indole concentration in normal and CKD model mice significantly decreased from 1.1 ng/mg to 0.5 ng/mg and 0.7 ng/mg to 0.4 ng/mg, respectively.

In order to assess the efficacy of antibiotics in clearing a niche for subsequent colonization with *E. coli*, we plated mice feces on media in aerobic or anaerobic conditions to detect bacteria. In figure 5f, the results showed that the number of mice fecal bacteria was greatly reduced 3 days after antibiotics treatment, indicating the efficacy of antibiotics to remove gut bacteria. After *E. coli* colonization for 3 days, the mice fecal bacteria increased significantly, indicating the success colonization of *E. coli*. At the same time, fecal bacteria were detected after 3 days of *E. coli* colonization and ISO treatment, and it was found that the ISO group mice had a similar number of fecal bacteria as *E. coli* colonization group mice, suggesting that the ISO group had a similar colonization of *E. coli*. These results suggest that the significant decrease in fecal indole concentration in the ISO group was not due to the difference in the amount of bacterial colonization between the groups. These data suggest that ISO inhibits indole production *in vivo*.

We found that the plasma IS concentration in CKD model mice was significantly reduced after treatment with ISO (80 mg/kg) by oral gavage for 4 weeks. We used plasma creatinine (Cr) to reflect the kidney function of each group mice. The circulating levels of IS and plasma Cr in the normal kidney function mice ± ISO and in CKD model mice ± ISO was also measured (Figure 5g, 5h and 5i). These results shown that ISO doesn’t improve kidney function and clearance of IS. Moreover, in mice with normal kidney function, ISO did not reduce circulating levels of IS.

### Deglycosylation of flavonoid glycosides is critical for their inhibitory effect on indole production

**Figure 6.**
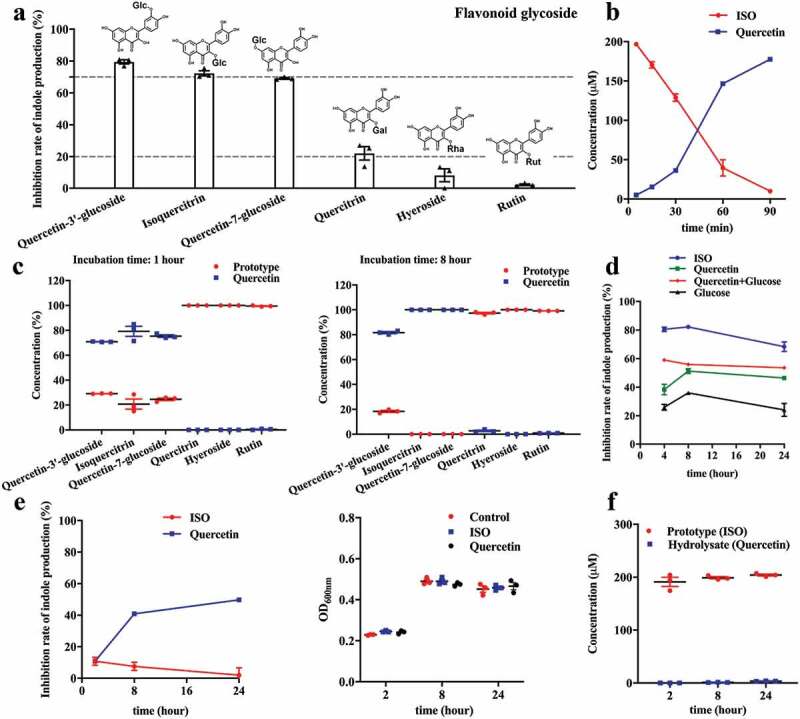
Deglycosylation of flavonoid glycosides is critical for their inhibitory effect on indole production. (a) The structure of flavonoid glycosides and the rate of indole production inhibition in *E. coli* after co-incubation with 200 μM flavonoid glycosides for 8 h. (b) The concentration of ISO and its aglycon, quercetin, after *E. coli* incubation with 200 μM ISO for 5 to 90 min. (c) The concentration of flavonoid glycosides and their aglycone, quercetin, after *E. coli* incubation with 200 μM flavonoid glycosides for 1 h (left) and 8 h (right). (d) The inhibition rates of *E. coli* co-incubated with 200 μM ISO, 200 μM quercetin, 200 μM glucose, or 200 μM quercetin plus 200 μM glucose on indole-production. (e) The inhibitory rates of ISO on indole production (left) and effect of ISO on OD_600nm_ (right) of *B. theta* co-incubated with 200 μM ISO or 200 μM quercetin for 2, 8, and 24 h. (f) The concentration of ISO and its hydrolysis aglycone, quercetin, after 200 μM ISO incubation with *B. theta* for 2, 8, and 24 h. All the data were normalized with OD or intracellular protein.

Glycosides are the primary form of flavonoids in nature. To investigate the effect of conjugated glycosides on the inhibitory effect of flavonoids, different kinds of flavonoid glycosides, including ISO, quercetin-3ʹ-O-glucoside, quercetin-7-O-glucoside, quercetin-3-O-rhamnoside (quercitrin), quercetin-3-O-galactoside (hyperoside), and quercetin-3-O-rutinoside (rutin) were tested. Glucoside forms of flavonol quercetin, including ISO, quercetin-3ʹ-O-glucoside, and quercetin-7-O-glucoside, are potent inhibitors (> 70%) of indole production. However, non-glucoside conjugates, such as quercetin-3-O-rhamnoside (quercitrin), quercetin-3-O-galactoside (hyperoside), and quercetin-3-O-rutinoside (rutin) were less potent inhibitors of indole production (Figure 6a).

*E. coli* has glucosidase activity and can hydrolyze the conjugated flavonol glucosides into aglycon and glucoside. Therefore, we further tested the ability of *E. coli* to hydrolyze the aforementioned flavonoid glycosides, leading to the release of aglycon and subsequent inhibition of indole production. As expected, we found that ISO was hydrolyzed to quercetin after incubation with *E. coli* (Figure 6b). The hydrolysis profiles of other flavonoid glycosides are shown in Figure 6c. The glucoside glycosides were hydrolyzed to different degrees after co-incubation with *E. coli* for 1 h, and were almost completely hydrolyzed after 8 h. However, the non-glucoside glycosides were not hydrolyzed by bacteria, and they still exist in prototype form (Figure 6c). Therefore, we postulated that the glycoside hydrolysis is critical for the inhibitory effects of flavonoid glycosides.

As the above results showed that ISO could be hydrolyzed to glucose and quercetin, we postulated that ISO exerts its effect via glucose and quercetin. In order to confirm this hypothesis, *E. coli* were directly incubated with 200 μM glucose plus 200 μM quercetin for 4, 8, and 24 h, and the inhibition rates were compared to 200 μM ISO (Figure 6d). In contrast to our hypothesis, the results demonstrated that the combination of glucose and quercetin did not exhibit similar inhibitory effects as ISO. Indeed, the effect of ISO was more potent than the combination of quercetin and glucose.

Based on these data, we hypothesized that the inhibitory effect of ISO on indole production might also be associated with its deglycosylation activity. Therefore, ISO may exert its effect in three possible ways. First, the deglycosylation activity of ISO consumes hydrogen (H) protons in the bacterial membrane, which leads to decreased bacterial membrane potential and decreased tryptophan transport. Second, the deglycosylation activity of ISO releases aglycon quercetin, which may inhibit complex I activity and decrease bacterial membrane potential and tryptophan transport. Third, the deglycosylation activity of ISO will also release glucose, which inhibits indole production through catabolite repression.

*Bacteroides thetaiotaomicron (B. theta)* also exists in the intestine and can produce indole. *B. theta* was incubated with ISO for 2– 24 h, and the inhibitory effect of ISO on indole production was evaluated. We found that the ISO-mediated inhibition of indole synthesis was abolished when co-incubated with *B. theta* (Figure 6e). However, quercetin was still able to inhibit indole production in *B. theta*, which is consistent with the effect of quercetin on *E. coli*. We hypothesized that ISO could not be hydrolyzed by *B. theta* and, therefore, could not inhibit indole production. Next, we evaluated the hydrolysis profile of ISO in *B. theta*. Consistent with our hypothesis, ISO was not hydrolyzed during co-incubation with *B. theta* (figure 6f). Therefore, ISO does not inhibit indole synthesis in *B. theta*, thereby supporting the hypothesis that glycoside hydrolysis is critical for the inhibitory effects of flavonoid glycosides.

### Structure-activity relationship of natural flavonoids on indole production

**Figure 7.**
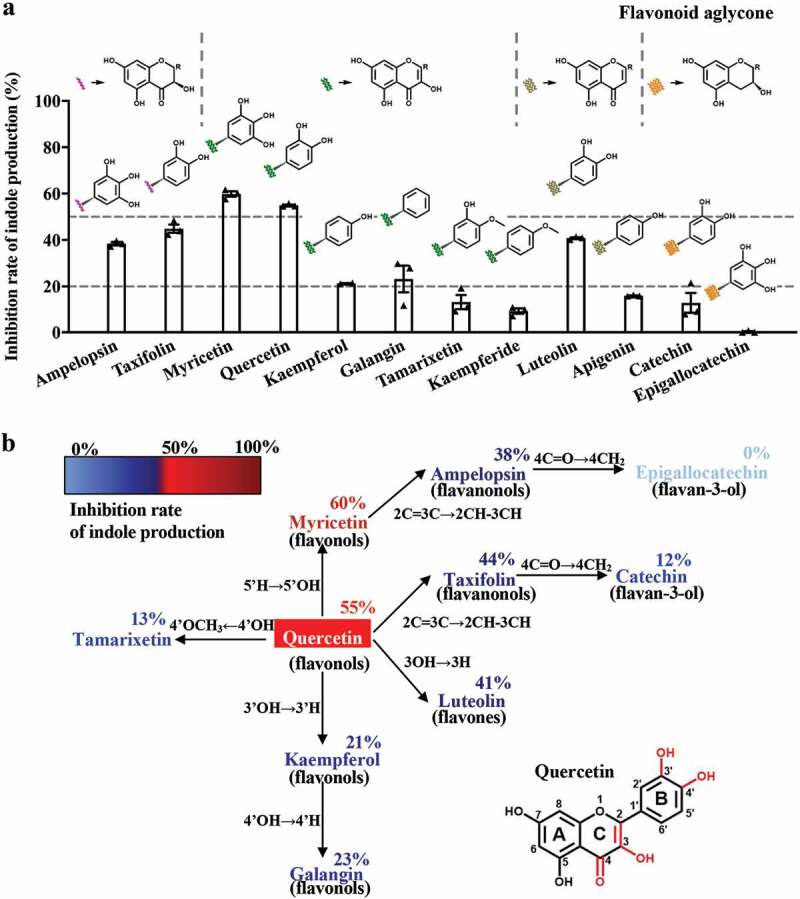
Structure-activity relationship of natural flavonoids with respect to indole production. (a) The structure of flavonoid aglycone and the rate of indole production inhibition in *E. coli* after incubation with 200 μM flavonoid aglycone for 8 h. (b) The addition or subtraction of functional groups based on quercetin and their inhibition rates on indole production.

The above data showed that ISO inhibits indole production after releasing its aglycone quercetin. We then evaluated the structure-activity relationships of other flavonoid aglycones. Our data show that the inhibitory potency of myricetin and quercetin exceeds 50% (Figure 7(a,b)), which is higher compared to other flavonol aglycones. The inhibitory potencies of kaempferol (with only one OH-group in the B-ring) and galangin (with no OH-group on B-benzene) were only 20%. These data indicate that the phenolic hydroxyl group is important for aglycone activity. Interestingly, the flavonoid aglycone with more phenolic hydroxyl groups showed increased inhibitory potency. When the para-OH of the B-ring phenolic hydroxyl group was methylated (tamarixetin and kaempferide), the inhibitory potency of the methylated aglycon decreased significantly.

In the flavonol aglycone molecule, C-3 is linked to an OH group. When the 3-OH group in the chromone moiety was absent (luteolin and kaempferide), the inhibitory potency decreased significantly, even in the presence of *o*-dihydroxy in the B-ring.

In addition, we were curious to learn whether the presence or absence of a 2,3-double bond would influence the inhibitory potency of aglycone. Therefore, we compared the inhibitory potencies of ampelopsin and taxifolin on indole production. As the inhibitory potencies of ampelopsin and taxifolin were lower than those of myricetin and quercetin, respectively, it is reasonable to conclude that the presence of the 2,3-double bond is also important for the inhibitory potency of flavonoid aglycone.

Furthermore, when the 4-oxo group was absent in the C-ring, the inhibitory potency of flavonoid aglycone was almost eliminated, thereby suggesting that the 4-oxo group in conjugation with a 2,3-double in the C-ring is crucial for the inhibitory effect of flavonoid aglycone.

Overall, the above data suggest that the following structural fundamentals of flavonoids are important for their indole-production inhibitory activity: the 4-oxo group in conjugation with a 2,3-double bond in the C-ring, the presence of *o*-dihydroxy in the B-ring, and the 3-OH in C-ring. These key functional groups play important roles in the inhibitory potency of flavonoid aglycone on indole production.

## Discussion

The gut microbiota play an important role in health and disease. In recent years, significant progress has been made in the treatment of human diseases by targeting the gut microbiota. Studies by Hazen and colleagues provide evidence for the hypothesis that targeting gut microbiota-dependent TMA and TMAO synthesis through microbial TMA lyase inhibition might serve as a new strategy to reduce host thrombosis risk.^1^

In CKD patients, insufficient kidney clearance leads to high levels of uremic solutes present in the blood. IS is a representative uremic solute that is retained in patients with CKD patients.^9^ Hemodialysis is commonly used to clear IS in CKD patients, but the outcome of hemodialysis is not satisfactory. Patients who undergo hemodialysis have a low quality of life, and their average 5-year survival rate is less than 50%.^44^ IS is not effectively removed by hemodialysis because it is tightly bound to plasma proteins. Kidney transplantation is currently the best treatment option for end-stage nephropathy; however, this strategy is limited by the shortage of kidney donors, the high cost, and the extremely high incidence of organ rejection after surgery.^45^

Due to the limitations of the current best treatment strategy, reducing the production of uremic solutes, including IS, represents a promising alternative method to slow CKD progression and increase the quality of life of patients. Uremic toxin adsorbents such as AST-120 can adsorb uremic toxin precursors in the intestines and decrease uremic toxins *in vivo*.^27^ However, adverse reactions such as constipation, nausea, and vomiting limited the use of AST-120. In addition, probiotics can reduce the production of uremic toxins by regulating the intestinal microenvironment,^46^ but the effects of probiotics are not specific.

Indole is generated by the gut microbiota-mediated tryptophan metabolic pathway, and TnaA is the only known enzyme which participate in this process in gut bacteria. In 2016, Sonnenburg and Fischbach explored the possibility of regulating the level of IS precursors in the gut via antibiotic treatment combined with re-colonization by a tryptophanase-deficient strain. They reported that modulation of the microbiota lowered the amount of indole produced in the gut, thereby reducing IS levels in circulation and presenting a potentially novel strategy to delay the progression of CKD.^11^ Their study showed that the circulating uremic toxin, IS, can be modulated via rational genetic manipulation of the gut microbiota. However, regulation of the gut microbiota metabolism pathway and inhibition of uremic toxin synthesis by small molecules has not yet been reported.

In this study, we have identified a natural small-molecule that significantly inhibits gut microbiota-mediated indole production without affecting bacterial growth. Furthermore, this small molecule shows a distinct action profile compared to antibiotics.

**Figure 8.**
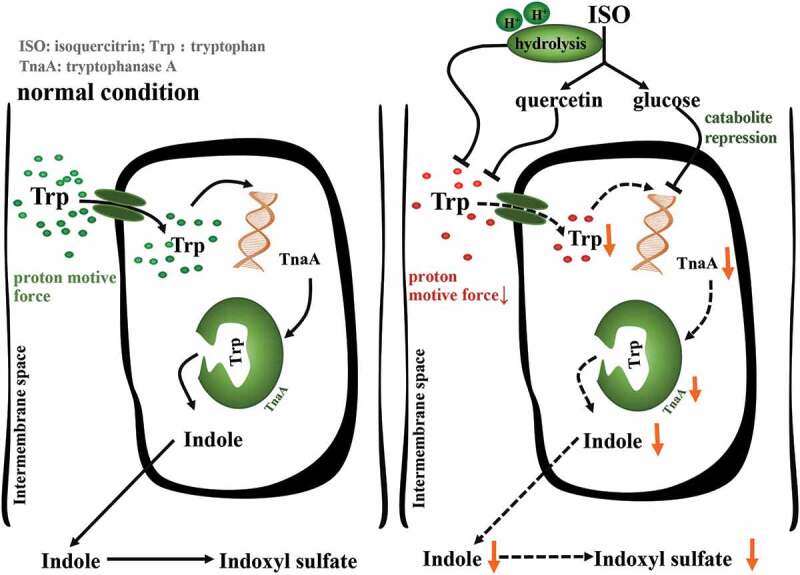
Schematic diagram of the action of ISO on indole production at the cellular level. The left panel displays bacterial biosynthesis of indole under normal conditions and the right panel displays bacterial biosynthesis of indole in the presence of ISO.

Tryptophan uptake by bacteria requires a synergistic effect of the H proton potential. We found that ISO can inhibit complex I activity, which is a part of the electron transport chain in the bacterial membrane, thereby weakening the H proton potential and reducing tryptophan transport into the bacteria. Subsequent experiments have shown that the presence of various electron acceptors attenuates the inhibitory potency of ISO on indole-production and tryptophan transport. In conclusion, ISO inhibits the establishment of H proton potential by regulating the gut bacteria electron transport chain, thereby inhibiting the transport of tryptophan and further reducing indole biosynthesis. Interestingly, ISO inhibits indole synthesis from three synergies, including the deglycosylation process, which may consume bacterial H protons as well as release aglycon quercetin, which inhibits complex I activity^47^ and releases glucose, which act through catabolite repression^48^ (Figure 8).

In this study, 200 μM ISO was used in most experiments because we found that at this concentration, ISO effectively inhibits indole synthesis without affecting bacterial growth. An adult has 1– 2 L of body fluid entering the colon via the valvular ceca every day. Therefore, we estimated that a CKD patient needs to consume 92– 185 mg (1.5 mg/kg – 3 mg/kg assuming a bodyweight of 60 kg) of purified ISO per day to maintain a final concentration of 200 μM in the intestinal fluid.

Research has shown that the proportion of the Enterobacteriaceae bacterial species is significantly increased in the intestines of patients with CKD. As a representative strain of the Enterobacteriaceae family, *E. coli* can produce indole eventually leading to an increase in uremic toxin IS in the patient’s plasma. We found that the effect of ISO is more significant when incubated with the bacteria in the exponential growth stage. In the early stage of CKD, the Enterobacteriaceae family will show a significant expansion, which may exhibit exponential growth characteristics. If CKD patients ingested ISO in the early stage of the disease, ISO may inhibit bacterial indole synthesis during the exponential growth stage, thus reducing the accumulation of uremic toxin IS.

Microbiologists have generally assumed that “the yield of cells is directly proportional to the amount of ATP produced”. But later it was also proposed that “the correlation between ATP content and biomass formation in bacteria is often poor due to maintenance energy requirements and other ways of non-growth energy dissipation.”^49^

It has been assumed that all of the energy from catabolism can be used for growth; however, bacteria also expend energy on functions that are not directly growth related.^49^ ATP is the energy currency of the cell, providing energy for a variety of biological processes such as enzymatic reactions, synthesis of macromolecules, active transportation of many substrates.^50,51^ When bacterial ATP production is interrupted, the bacteria will maintain its growth at the expense of other non-essential functions. Just as we found in the present study, ISO could intervene the energy formation process of bacteria but did not impact cell growth. Moreover, ISO could inhibit the transportation of Trp and therefore decrease indole production. Based on these observations, we proposed that when bacterial ATP production is interrupted, the bacteria will maintain its growth at the expense of other non-essential functions such as transporting of amino acids, which will keep the necessary energy for bacteria growth.

In conclusion, our investigation demonstrates that it is possible to inhibit gut microbial indole production with natural components, which is different from the action of antibiotics. Moreover, our study showed that targeting gut bacteria may represent the first step in modulating indole production in the gut and may contribute to delaying CKD progression.

## Methods

### Bacteria culture

The strain separated from healthy human feces was identified as *E. coli* by Sangon Biotech (Shanghai, China) based on 16S rDNA sequence analysis. The isolated strain shares 100% sequence similarity to the *E. coli* strain NCTC8623. *E. coli* was streaked onto LB plates and incubated at 37°C in an anaerobic workstation (AW200SG, Electrotek, UK), which was filled with mixed nitrogen (10% H_2_, 10% CO_2_, 80% N_2_). A single colony was inoculated into 5 mL LB medium and grown overnight. The cultures were diluted 1:100 using LB for the subsequent experiments. A 500 μL aliquot was seeded in a 1.5 ml Eppendorf tube and repeated in triplicate. *E. coli* cells were cultured in tubes in the presence or absence of ISO (200 μM, 100 μM, and 50 μM) at 37°C under anaerobic conditions. The OD_600_ was measured using a microplate reader (PerkinElmer, USA). *B. theta* ATCC 29,148 was obtained from Guangdong Microbial Culture Center (Guangdong, China) and grown in tryptic soy agar (TSA) plus 5% (volume/volume) sterile defibrinated sheep blood. Other preparations were prepared as above. After co-incubated with or without 200 μM ISO for 8 h, the bacteria were diluted in an appropriate gradient and plated on LB solid plate. Anaerobic culture for 24 h and the number of colonies were quantified.

### Indole analysis

Culture supernatants (100 μL) were thoroughly mixed with 300 μL acetonitrile and centrifuged at 12,000 rpm (4°C) for 10 min. The supernatants were analyzed with a HPLC-FLD (Waters, USA) using a 250 mm × 4.6 mm C18 column (Alltima, USA) and elution with acetonitrile (solvent A) and 200 mM ammonium acetate (pH 4.5; solvent B) as the mobile phases at a flow rate of 1 mL/min, with a column temperature of 35°C. The eluting conditions were 70% (A) and 30% (B). Based on this condition, the indole retention time was 4.4 min, and the fluorescence excitation and emission wavelengths were set at 270 nm and 340 nm, respectively.

### Collection and incubation of mixed gut bacteria

SD rats and C57BL/6 mice were obtained from Vital River Laboratory Animal Technology Corp. (Beijing, China). All animal regulations were approved by the Animal Care and Use Committee of Nanjing University of Chinese Medicine. The rats and mice were permitted free access to purified water and rat-chow. Fresh feces and cecal contents were obtained from 8-week-old healthy rats or mice. All samples were processed immediately, mixed 1:4 (mg/mL) with aseptic physiological saline, then homogenized completely in a vortex oscillator (Grant Instruments, EU) for 2 min. After centrifugation (200 g, 5 min, 4°C), the supernatants were used as the intestinal bacterial mixture after dilution with LB to reach an OD_600_ of 0.1.

#### Enzymatic reaction

The TnaA enzymatic reaction was performed as described earlier^52,53^ with minor changes. *E. coli* were collected by centrifugation at 10,000 rpm (4°C) for 3 min and resuspended in 0.5 mL (equal to the culture volume) phosphate buffer solution (0.2 M; pH 7.2). Bacteria were frozen at −80°C and thawed in a refrigerator. This freeze thaw cycle was repeated three times, then bacteria were further lysed by ultrasonification at 500 W with a 3 s on, 10 s off pulse rate. This was repeated 5 to 7 times and cell lysates were subsequently centrifuged at 4,500 rpm (4°C) for 10 min. The supernatant was incubated with 0.1 mL pyridoxal phosphate (100 μg/mL) for 10 min under anaerobic conditions at 37°C. Subsequently, 0.1 mL tryptophan (0.5 M) was added to the samples which were left to incubate at 37°C for 30 and 60 min. Next, triple acetonitrile was added and mixed thoroughly to terminate the reaction and the samples were centrifuged at 12,000 rpm for 10 min. The supernatants were used to measure the concentration of indole by HPLC-FLD. As reported in the literature,^43,53,54^ the units of TnaA enzyme activity were calculated as follows:

arbitrary units (AU) = 1 ug indole produced/min/OD_600nm_ of bacteria.

Enzyme activity was normalized against bacteria with an OD value of 1.

#### Tryptophan analysis

UPLC-TQ/MS was used to quantify tryptophan and Trp-D_8_ (C/D/N Isotopes Inc., Quebec, Canada) in intracellular or culture supernatants. Centrifugation was carried out at 10,000 rpm for 5 min at 4°C to harvest the bacteria. The cell pellet was then washed three times with phosphate buffered saline (PBS), and lysed by ultrasonication after resuspension in 100 μL PBS. The culture supernatants and bacterial lysates were added to triple acetonitrile to precipitate the proteins, mixed thoroughly, and centrifuged at 12,000 rpm for 10 min. The supernatants were then analyzed by UPLC-TQ/MS to detect tryptophan concentrations.

Chromatographic analysis was performed on an Acquity UPLC BEH Chromatographic column (100 mm × 2.1 mm, 1.7 μM, Waters, Ireland) using an Acquity UPLC TM system (Waters, USA) for tryptophan detection. The mobile phase consisted of 0.15% (v/v) formic acid containing 5 mM ammonium formate and 5 mM ammonium acetate (solvent A) and acetonitrile containing 1 mM ammonium formate and 1 mM ammonium acetate (solvent B), with a flow rate of 0.4 mL/min. The elution conditions were set at 6% (A; v/v) and 94% (B; v/v) with a 2 μL injection volume. The temperatures of the column and auto-sampler were 35°C and 4°C, respectively.

Mass spectrometry analysis was performed using selective reaction monitoring (SRM) on a triple quadrupole mass spectrometer (TQ/MS; Waters, USA). The parameters for the test were as follows: capillary voltage, 3.0 kV; source temperature, 150°C; cone gas flow, 50 lh; desolvation temperature, 500°C; desolvation gas flow, 1000 l/hour; and collision gas flow 0.15 mL/min. The optimal detection conditions for the compounds are shown in Table 1.

**Table 1.** Optimal detection conditions for compounds by UHPLC-TQ/MS.

Compound Name	Ionization Mode	Parent ion /m·z^−1^	Daughter ion /m·z^−1^	Cone voltage /V	Collision energy /V	Retention Time /min
IS	ES^−^	212.0319	79.9677	22	24	2.06
Chloramphenicol	ES^−^	321.0958	151.7981	24	18	3.38
Tryptophan	ES^+^	205.0958	146.0958	16	18	1.51
Tryptophan-D_8_	ES^+^	213.7981	141.3155	100	24	1.51

### Analysis of membrane potential

Membrane potential analysis was performed as previously described^55^ and with minor changes. After incubation, 500 μL of cell culture at an OD_600_ of 0.2 were mixed with 500 μL PBS and 1 mM EDTA was added at 37°C for 2 min to weaken the outer membrane. The cells were then stained with 60 μM DioC_2_(3) for 15 min. In addition, 5 μM CCCP was added as a control prior to staining for 5 min, resulting in the loss of the membrane potential. During the staining process, all samples were protected from light. After staining, 20 μL of the bacterial suspension was transferred onto a microslide and observed under a fluorescence microscope (OLYMPUS BX53, Japan).

### Respiratory chain complex activity, NADH/NAD^+^, ATP, Cr and total protein content analysis in E. coli

*E. coli* was incubated with 200 μM ISO for 1 h and 8 h. Centrifugation was carried out at 10,000 rpm for 5 min to collect bacteria. The bacterial pellet was then lysed by ultrasonication after resuspension in 100 μL PBS. Respiratory chain complex activities were measured using a complex I assay kit (Suzhou Comin, Cat# FHTA-2-Y), the total protein content of bacteria was measured using a Bradford Assay Kit (Thermo Fisher Scientific, Cat# 23,200). After lysed, the supernatants were collected by centrifugation at 10,000 rpm for 5 min, and then NADH/NAD^+^ was measured using an NAD/NADH assay kit (Abcam, Cat# ab176723). *E. coli* was incubated with 200 μM ISO for 0.25– 2 h. Intracellular ATP was measured in the collected bacterial pellets using an ATP Assay Kit (Beyotime, Cat# S0026). The plasma creatinine (Cr) was measured using a creatinine assay kit (Nanjing Jiancheng Bioengineering Institute, Cat# C011-1-1).

#### Mice

All procedures were approved by the Institutional Animal Care and Use Committee of the Nanjing University of Chinese Medicine. An adenine-induced CKD model was established in C57BL/6 male mice. The control mice were administered 0.5% (weight/volume) carboxymethyl cellulose (CMC-Na,) by oral gavage (0.1 mL per 10 g body weight) daily. The CKD model mice were administered 50 mg/kg adenine, which was suspended in 0.5% (weight/volume) CMC-Na by oral gavage (0.1 mL per 10 g body weight) daily. After oral administration for 28 days, we found that the body weight of the model mice was significantly reduced compared to the control group. Mouse intestinal bacteria were removed using an antibiotic cocktail, as previously described.^11^ HPLC-FLD analysis showed that after 3 days, fecal indole was undetectable (Figure 5c). *E. coli* were cultured overnight, bacteria were collected, resuspended in LB media (10^8^ CFU), and used as transplant material. Mice were inoculated daily with fresh transplant material (0.1 mL per 30 g body weight) and ISO 80 mg/kg (0.1 mL per 10 g body weight) by oral gavage for 3 to 6 days. Fresh feces were collected and the concentration of indole was detected by HPLC-FLD. The bacteria transplantation protocol was performed in the same way for normal mice.

Indole content was measured in mouse feces as follows: fresh feces were collected and mixed 1:4 (mg/mL) with aseptic physiological saline. After centrifugation (10,000 rpm, 5 min), the supernatants were thoroughly mixed with triple acetonitrile and centrifuged at 12,000 rpm for 10 min, and the supernatants were analyzed by HPLC-FLD.

Mice were randomized into 3 groups: 6 in the normal group, 10 in the adenine-induced CKD model group, and 10 for the ISO-treated group. After treatment with 80 mg/kg ISO for 4 weeks, blood was collected 24 h after the last scheduled treatment. After centrifugation (3,500 rpm, 10 min), plasma was collected and mixed in a 10:1 (v/v) ratio with 200 μg/mL of chloramphenicol as an internal standard, then mixed with triple acetonitrile and centrifuged at 12,000 rpm for 10 min. The supernatants were then analyzed by UHPLC-TQ/MS.

#### IS analysis

UPLC-TQ/MS was used to quantify tryptophan in intracellular and culture supernatants. Chromatographic analysis was performed on an Acquity UHPLC BEH C18 Chromatographic column (100 mm × 2.1 mm, 1.7 μM, Waters, Ireland) using an Acquity UPLC TM system (Waters, USA) for IS detection. The mobile phase consisted of 0.15% (v/v) formic acid containing (solvent A) and acetonitrile (solvent B), with a flow rate of 0.4 mL/min. The elution conditions were set to (0 min – 1.0 min) 14% – 21% B, (1.0 min – 4.0 min) 21% – 40% B, (4.0 min – 4.2 min) 40% – 95% B, (4.2 min – 5.0 min) 95% B (5.0 min – 5.2 min) 95% – 14% B, (5.2 min – 5.5 min) 14% B. The injection volume was set at 2 μL. Other detection methods include tryptophan analysis. The optimal detection conditions for the compounds are shown in Table 1.

### Flavonoid metabolism analysis

*E. coli* or *B. theta* was incubated with 200 μM flavonoids for 0– 8 h. The cultures were mixed with triple acetonitrile and centrifuged at 12,000 rpm for 10 min. The supernatants were subsequently analyzed by UPLC. Chromatographic analysis was performed on an Acquity UPLC C18 Chromatographic column (100 mm × 2.1 mm, 1.7 μM, Waters, Ireland) using an Acquity UPLC TM system (Waters, USA). The mobile phase consisted of 0.1% (v/v) formic acid (solvent A) and acetonitrile (solvent B), with a flow rate of 0.4 mL/min. The gradient elution was as follows: 0– 3 min, 83% solvent A; 3– 5 min, 83% – 70% solvent A; 5– 7 min, 20% solvent A; 7– 8 min, 83% solvent A with an injection volume of 1 μL. The temperatures of the column and auto-sampler were 35°C and 4°C, respectively, and the detection wavelength was set to 360 nm.

#### Data analysis

GraphPad Prism version 8.0 (GraphPad Software, San Diego, CA) was used for statistical analysis. All measurements were repeated in triplicate and performed under the same experimental conditions. Data are expressed as the mean ± standard error mean (SEM). The nonparametric Mann-Whitney test was used to analyze the differences between groups, a *P* value < .05 was considered to be statistically significant, and a *P* value < .01 was accepted as very statistically significant.
